# Inclusive-fitness logic of cooperative breeding with benefits of natal philopatry

**DOI:** 10.1098/rstb.2013.0361

**Published:** 2014-05-19

**Authors:** Geoff Wild, Cody Koykka

**Affiliations:** Department of Applied Mathematics, The University of Western Ontario, London, Ontario, Canada N6A 5B7

**Keywords:** alloparental care, delayed dispersal, helpers-at-the-nest, kin selection, social evolution, theory

## Abstract

In cooperatively breeding species, individuals help to raise offspring that are not their own. We use two inclusive-fitness models to study the advantage of this kind of helpful behaviour in social groups with high reproductive skew. Our first model does not allow for competition among relatives to occur but our second model does. Specifically, our second model assumes a competitive hierarchy among nest-mates, with non-breeding helpers ranked higher than their newborn siblings. For each model, we obtain an expression for the change in inclusive fitness experienced by a helpful individual in a selfish population. The prediction suggested by each expression is confirmed with computer simulation. When model predictions are compared to one another, we find that helping emerges under a broader range of conditions in the second model. Although competition among kin occurs in our second model, we conclude that the life-history features associated with this competition also act to promote the evolutionary transition from solitary to cooperative breeding.

## 1. Introduction

Helpful behaviour takes many forms in vertebrates [1] but some of the most interesting instances come from species that breed cooperatively. In cooperatively breeding species, individuals help to raise offspring produced by others in their group. These species offer researchers a valuable opportunity to study helping in an arena where its substantial personal costs can be linked easily to the improved reproductive success of a neighbour [2,3].

Hamilton's rule [4] is typically used to explain the adaptive significance of cooperative breeding. Inclusive fitness, in this case, is split into two parts: direct fitness, which reflects the production of descendant kin, and indirect-fitness, which reflects the production of non-descendant kin [5,6]. Hamilton's rule, then, predicts that cooperative breeding will be favoured when the net direct-fitness cost of helping is outweighed by the net indirect-fitness benefit.

Work on cooperative breeding is not typically concerned with evaluating the correctness of Hamilton's rule. Instead, the focus is placed on understanding the relative importance of the rule's direct and indirect components [7]. Some authors have argued that ecological constraints (e.g. low probability of successful, independent breeding) mean that helping changes the direct fitness of the helper by only a small amount. These authors point to the net indirect-fitness benefit of helping as its principal evolutionary driver [8–10]. Other authors have taken a different stance and argued that inheritance of a breeding territory (especially inheritance of a high-quality breeding territory) can translate into large, direct-fitness gains for those who provide help at their natal site. Under this alternative view, it is the direct ‘benefits-of-philopatry’ that act as the main incentives to help [7]. In this paper, we emphasize the benefits-of-philopatry perspective and model the emergence of cooperative breeding when breeding territories can be passed from parent to offspring.

We are not the first to model cooperative breeding with benefits-of-philopatry. Pen & Weissing [11] used an inclusive-fitness model to demonstrate how the direct-fitness benefits of territory inheritance depend on life-history details in species. Their model, however, did not address the possibility that an individual may eventually compete with the offspring it helps to rear. Such competition among territorial cooperative breeders is likely to be severe [12]. In fact, theoretical work by Leggett *et al*. [13] has shown that the selective advantage of cooperative breeding is diminished when the possibility of territory inheritance leads to competition among kin. In Leggett *et al*.'s model, related individuals competed against one another for territory ownership on an equal basis. Here, we extend their theoretical results to the case in which there is a rigid hierarchy among related competitors. We simply ask, under what conditions will cooperative breeding emerge?

To answer our question we build two inclusive-fitness models that both pay careful attention to population dynamics. In this way, our approach differs from many other mathematical models of cooperative breeding (e.g. [10,14,15]), because it ensures that model predictions are consistent with ecologically reasonable scenarios like bounded population growth (this point was originally made by Pen & Weissing [11]). The attention we pay to population dynamics also clarifies how the fitness changes that immediately follow a helpful social interaction might be accommodated, later, by additional changes in the fitness of an actor's relatives—especially fitness reductions that result from increased local competition.

Although our results can be framed using detailed population genetics, we provide a decidedly non-technical presentation that highlights the importance of Hamilton's contribution to evolutionary biology. With our first model, we recover the predictions of Pen & Weissing [11], but our predictions change with the second model. Our second model allows for local competition among siblings. Remarkably, we find that a broader range of ecological conditions support cooperative breeding in the second model, even though competition between relatives enters into consideration.

## 2. Population dynamics

We begin our modelling effort by considering the dynamics of a population that does not breed cooperatively. The population in question is assumed to be made up of diploid, simultaneous hermaphrodites. We have chosen to model individuals as hermaphrodites for mathematical convenience.

At the beginning of a given season, we find some number of non-breeding floaters, and breeders without attendant helpers.

Each breeder produces exactly one offspring per season through female function. To produce an offspring through female function, a given breeder mates with another individual who makes the male contribution to the offspring. The mate in question is chosen uniformly at random from the population of breeders. Thus, each breeder will also produce, on average, one offspring per season through male function.

An offspring is born on the territory belonging to its ‘mother’ (i.e. the parent who made the maternal contribution). We assume that each offspring survives with probability *p* (all notation is summarized in table 1).

**Table 1.** Summary of mathematical notation used in the main text.

symbol	explanation
*p*	the viability of an offspring born to a breeder without help
*q*	the viability of an offspring born to a breeder with help
*σ*	the probability with which a breeder survives from one season to the next
*s*	the probability with which a helper survives from one season to the next
*t*	the probability with which a floater survives from one season to the next
*v*	the reproductive value of a ‘normal’ individual at birth (i.e. a normal surviving offspring)
*v*_f_	the reproductive value of a ‘normal’ floater (model II only)
*v*_i_	the reproductive value of a ‘normal’ individual who inherited a territory (model II only)
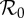	the basic reproduction number, defined as the expected number of new breeders produced by a given breeder in a population of low density

Following reproduction, breeders may die. We use *σ* to denote the probability with which breeders survive to the next season. When a breeder survives, it retains its breeding territory and its surviving offspring (the one it produced through female function) disperses, becoming a floater in the process. When a breeder dies, the events that follow will depend on the model scenario that interests us at the moment. In the first scenario (model I), we assume that a surviving offspring disperses when its parent dies and that the parent's territory disappears. In this first scenario, natal dispersal behaviour is independent of parental survival, and so there is no scope for an offspring to take over its natal territory. In the second scenario (model II), we assume that the territory of a parent that dies is inherited by its surviving offspring from that season; if no surviving offspring is present, then the territory disappears.

Each floater survives from one season to the next, independently, with probability *t*. Floaters that survive then become breeders with some probability that decreases with increasing total density of breeders. Specifically, we assume that the probability with which a surviving floater establishes itself as a breeder in a given season decreases with increasing density of breeders (see electronic supplementary material, sections S1 and S2). Of course, one minus the probability of establishment gives the probability with which a surviving floater fails to establish itself as a breeder in a given season.

The description above implies that a territory found in one season is found again the following season with probability *σ* in model I and with probability *σ* + (1*−σ*)*p* in model II. Once established, then, a territory remains intact for an average of 1/(1 *− σ*) seasons in model I and an average of 1/[1 *−* (*σ* + (1 *− σ*)*p*)] seasons in model II. In model I, a territory produces new floaters at a rate of *p* per season. By contrast, a territory in model II produces new floaters at a rate of *σp* per season. In both model scenarios, each floater needs only survive one season (probability *t*) in order to become established as a breeder when population densities are low. It follows that, at low population densities, each breeding territory produces 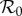 new breeding territories, where2.1
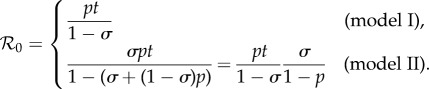
The quantity 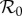 is often referred to as ‘the basic reproduction number’ in population biology. Although it has been denoted 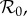 the basic reproduction number should not be confused with measures of relatedness.

**Figure 1.**
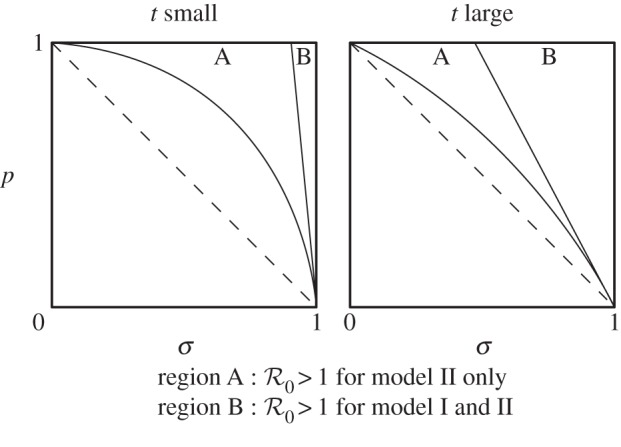
The region of parameter space supporting 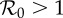 varies with the values taken by the probability parameters *p*, *σ*, and *t*. In region A 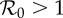 holds for model II only, whereas 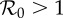 holds for both models I and II in region B. We find that decreasing *t* decreases the area of both regions A and B, and increasing *t* has the opposite effect. In fact, as *t* approaches zero the area of both regions vanishes, and as *t* approaches 1 the total area of both regions tends to 1/2 (i.e. the area in the unit square above the dashed line, *p* + *σ* = 1). Of course, for 0 < *t* < 1 both regions A and B lie above the dashed line. Thus, for 0 < *t* < 1 we conclude that 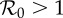 implies *p* + *σ* > 1(see the electronic supplementary material, sections S1 and S2).

It is possible to show that population extinction is avoided whenever 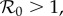 and that breeders and floaters achieve stable, positive equilibrium densities, respectively (see the electronic supplementary material, sections S1 and S2). It is also possible to show that 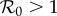 in model I implies that the analogous condition holds for model II; the converse, however, is not true (figure 1).

## 3. Advantage of delayed dispersal with helping

Cooperative breeding systems are quite varied. In some cases, cooperative breeders exhibit high reproductive skew with non-breeding helpers directing their help to a small number of dominant breeders, while in other cases reproduction is more evenly distributed [16]. It is the former case (‘helpers-at-the-nest’) that we model here. Specifically, we develop conditions for the selective advantage of an individual who delays natal dispersal for one season to help its mother rear the offspring she will next produce through female function.

We assume that the presence of a helper changes the probability of newborn survival from *p* to *q*. Helpers can improve offspring survival *q* > *p*, however our results do not require *q* > *p*. In some cooperatively breeding species, breeders reduce their level of investment in parental care in response to help [17,18], and so *q* ≤ *p* is also biologically reasonable.

Helpers in our model survive from one season to the next with probability *s*. While helper survival may be greater than that of floaters because of parental nepotism and familiarity with local conditions [19], we make no specific assumptions about the relative sizes of *s* and *t*. We do, however, assume that a helper inherits the territory of its mother in the event of her death. For model II, we assume that territory inheritance by a helper implies dispersal by a surviving newborn. In other words, experienced helpers inherit territories ahead of their inexperienced siblings in model II. In both models, surviving helpers that do not inherit a territory after one season disperse, becoming floaters in the process.

Below, we fix attention on an individual who behaves in a ‘deviant’ helpful manner, rather than in a ‘normal’ non-helpful manner. We then determine how the focal individual's inclusive fitness in a population at equilibrium changes as the result of its deviant behaviour. As the reader will see, our calculations assume that all relatives (including descendants) of the focal individual behave in a normal way. Technically speaking, we are assuming that the tendency to delay dispersal and help is owing to the presence of a rare allele at a single, autosomal locus and that the phenotypic effect of this rare allele is small (weak selection). In fact, the predictions we derive are equivalent to those made by detailed population-genetic models that make these same basic assumptions (see the electronic supplementary material, sections S3 and S4).

### (a) Model I

We consider an individual (the focal individual) who has decided to remain on its natal site to help its mother (recall that, had its mother not survived, then dispersal would have been automatic). We ask, how does the inclusive fitness of this individual change as a result of its decision? Before we start measuring change in inclusive fitness, though, we need to ensure changes will be commensurable by settling on a ‘common currency’. It turns out that any correct choice of common currency must reflect an individual's genetic contribution to the population in the very distant future, in other words an individual's reproductive value [20–22]. It is enough for now to point out that we can, without the loss of generality, set the reproductive value of a normal individual at birth (denoted *v*) equal to 1.

We now focus on changes to the indirect component of an actor's inclusive fitness. Because the focal individual has decided to remain on its natal site, its mother is able to produce an offspring through female function with probability *q*, rather than probability *p*. As a breeder chooses a new mate each season the focal individual and the offspring in question are half-siblings, related to one another by a factor of one-quarter. It follows that the focal individual's actions have immediately changed the indirect component of its inclusive fitness by an amount equal to (1/4)(*q* − *p*) *· v* = (1/4)(*q* − *p*) > 0. If *q* > *p*, then this term counts as an indirect-fitness benefit.

Turning our attention now to changes in direct fitness, we note that when both the focal individual and its mother survive (probability *s*σ*) the focal individual becomes a disperser. In this case, the focal individual (who is related to himself/herself by a factor of one) receives a normal direct-fitness pay-off *v* = 1. The direct-fitness pay-off, however, is exactly what the focal individual would have received had it behaved in a normal manner to begin with. Thus, with probability *σ*s*, the direct-fitness component of the focal individual's inclusive fitness experiences zero change.

If the focal individual survives and its mother does not (probability *s*(1 *− σ*)), then it will immediately inherit a territory. As a result, the focal individual expects to produce *p*/(1 *− σ*) offspring through male function and *p*/(1 *− σ*) offspring through female function over its lifetime, for a direct-fitness pay-off equal to (1/2) *· v ·* 2*p*/(1 *− σ*) = *p*/(1 *− σ*). It follows that, with probability *s*(1 *− σ*), the focal individual's behaviour changes its direct fitness by an amount equal to (*p*/(1 *− σ*) *− v*) = (*p*/(1 *− σ*) *−* 1) > 0.

In the case where the focal individual does not survive (probability 1 *− s*), it of course cannot produce any offspring of its own. Consequently, with probability 1 *− s*, the focal individual's direct fitness changes by an amount equal to (0 *− v*) = −1 as a result of its deviant behaviour.

Summing all changes to direct and indirect fitness (we can do this because we have used a common currency), we arrive at an expression for the overall inclusive-fitness effect of the focal individual's decision3.1
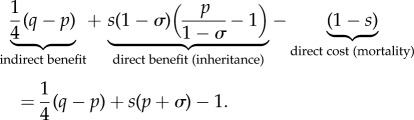


**Figure 2.**
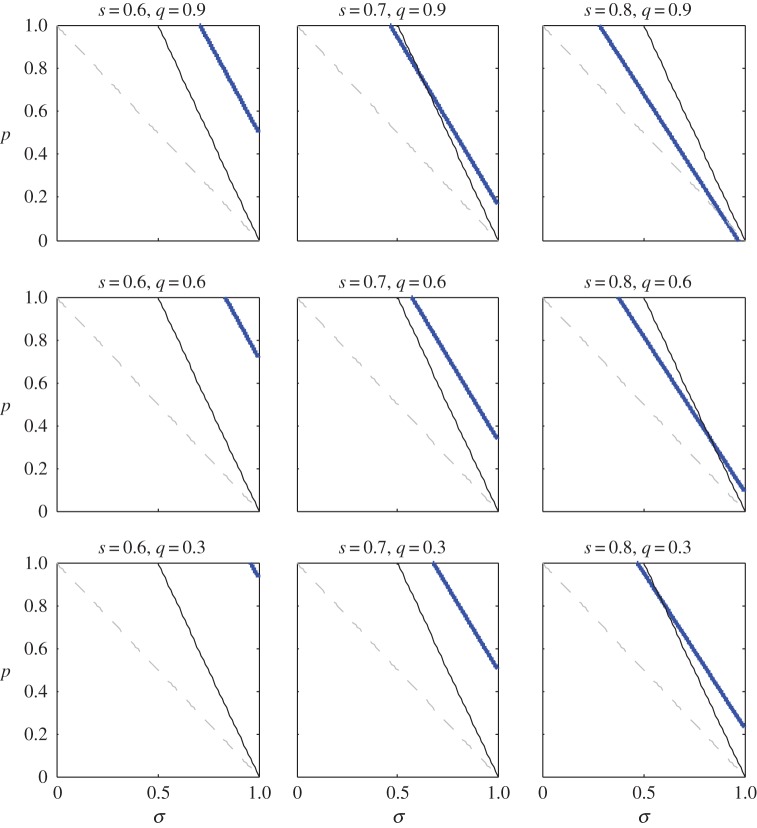
The region of ‘normal’ parameter space, *σ* and *p*, that supports cooperative breeding for various values of ‘deviant’ parameters *s* and *q*. Area above the blue line corresponds to a selective advantage for helping; i.e. the expression in (3.1) is positive. For comparison, we have superimposed the blue line on the relevant information from figure 1. Recall that, in order for 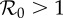 in model I, *σ* and *p* must fall above the solid black line (*t* = 0.5 shown). In addition, 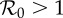 implies that the pair (*σ*, *p*) occurs above the dashed line. Overall, we see that increasing *s* or *q* (or both) increases the size of the region of parameter space that supports cooperative breeding.

An expression equivalent to (3.1) has previously been derived by Pen & Weissing [11] (their condition 25). When the expression in (3.1) is positive (respectively, negative), helping is favoured (respectively, disfavoured) by selection. Intuitively, for fixed values of the life-history parameters *σ* and *p*, we see that increasing deviant survival parameters *q* or *s* serves to increase the advantage of helping (figure 2).

### (b) Model II

In model II, the life history of a normal individual is more complicated than it was in model I. Consequently, we must elaborate on our description of reproductive value for model II.

In model II, a normal individual becomes a breeder either because it inherits a territory or because it establishes a new territory after being a floater for some period of time. Let *v*_f_ denote the reproductive value of a normal floater, and let *v*_i_ denote the reproductive value of a normal individual who inherits a territory. If *v* again denotes the reproductive value of a normal individual at birth, we must have *v* = *σv*_f_ + (1 *− σ*)*v*_i_. An established breeder in model II expects to produce (1/2) *·* 2*p*/(1 *− σ*) offspring over the course of its life, and so *v*_i_ = *v ·* (1/2)*·*2*p*/(1 *− σ*). It follows that the previous equation can be rewritten as *v* = *σv*_f_ + (1 *− σ*)*v*(*p*/(1 *− σ*)), and if we again set *v* = 1, we find *v*_f_ = (1 *− p*)/*σ*.

As before, we fix attention on an individual who has decided to remain on its natal site to help its mother and we ask how has this decision changed the focal individual's inclusive fitness?

The focal individual's deviant behaviour once again changes its indirect fitness. However, more care is required in model II in order to determine the amount and the sign of the change, because a helper's actions in this model can affect newborns in more than one way. If the focal individual survives and the breeder dies (probability *s*(1 *− σ*)), then the helper will displace a surviving newborn, sending it off to become a floater instead of allowing it to inherit the territory. In this case, the surviving newborn has a reproductive value *v*_f_ instead of *v*_i_. It follows that, with probability *s*(1 *− σ*), the indirect-fitness change amounts to (1/4)(*qv*_f_ − *pv*_i_).

**Figure 3.**
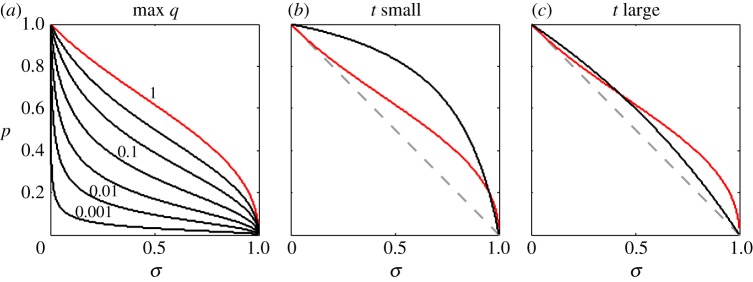
The region of ‘normal’ parameter space consistent with a cost interpretation of the first indirect-fitness term of the expression in (3.2) from model II. (*a*) The contours whose height corresponds to the largest *q* that could be consistent with a cost interpretation of the aforementioned term. The contour of height one has been highlighted in red, as it is the largest possible value *q* may take. For comparison, we have superimposed the red curve on the relevant information from figure 1. Recall that, in order for 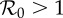 in model II, *σ* and *p* must fall above the solid black curve (*t* = 0.2 in (*b*), and *t* = 0.66 in (*c*)). In addition, 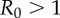 implies that the pair (*σ*, *p*) occurs above the dashed line. Overall, we see that the first indirect-fitness term of the expression in (3.2) is *not* necessarily a cost in the small region above the black curve and below the red curve.

Let us take a moment to consider the term (1/4)(*qv*_f_ − *pv*_i_) in more detail. As the previous discussion suggests, this term represents the effect of local competition among kin. In order for it to be counted as a cost, though, it must be negative; in other words, *q* cannot be too large (*q* < *pv*_i_/*v*_f_). Figure 3*a* shows how the largest value of *q* consistent with a cost interpretation changes as a function of *σ* and *p*. Of course, we always have *q* < 1, and so any combination of *σ* and *p* above the red line in figure 3*a* implies kin competition is costly.

We cannot forget that survival parameters *σ* and *p* are constrained by 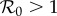. If we superimpose figure 3*a* on figure 1, we see that the cost interpretation of (1/4)(*qv*_f_ − *pv*_i_) can only fail in a narrow region of parameter space: that region above the solid black line but below the red line in figure 3*b,c*. We contend, therefore, that (1/4)(*qv*_f_ − *pv*_i_) is most likely an indirect-fitness cost. In fact, for sufficiently small *t* (i.e. for cases where floaters occupy harsh, marginal habitat) we are virtually guaranteed that (1/4)(*qv*_f_ − *pv*_i_) counts it as a cost (figure 3*b*).

Returning now to our development of the indirect-fitness effects, if either the focal individual dies (probability 1 *− s*), or if the focal individual and the parent both survive (probability *σs*), the newborn will not be affected by the focal individual's decision beyond the initial change in survival. In this former case, the newborn inherits a territory or becomes a floater just as any other normal newborn would. In the latter case, the newborn's success is equal to that of any normal floater. Therefore, with probability (1 *− s*) the indirect-fitness change amounts to (1/4)(*q* − *p*) *· v* = (1/4)(*q* − *p*), and with probability *sσ* the indirect-fitness change amounts to (1/4)(*q* − *p*) *· v*_f_.

The focal individual's actions only change its direct fitness in two ways. First, the focal individual dies with probability (1 *− s*), changing its direct fitness by an amount equal to *−v*_f_. If the focal individual survives and inherits its natal territory (probability *s*(1 *−σ*)), its direct fitness increases by an amount equal to *v*_i_ − *v*_f_. If the individual survives but does not inherit a territory, it disperses and its direct fitness is unchanged.

Summing all the changes to direct and indirect fitness, we find the inclusive-fitness effect of the focal individual's decision is3.2
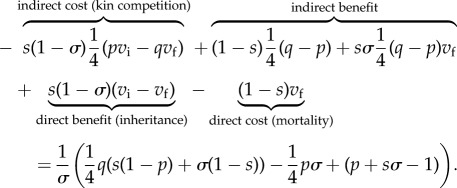


**Figure 4.**
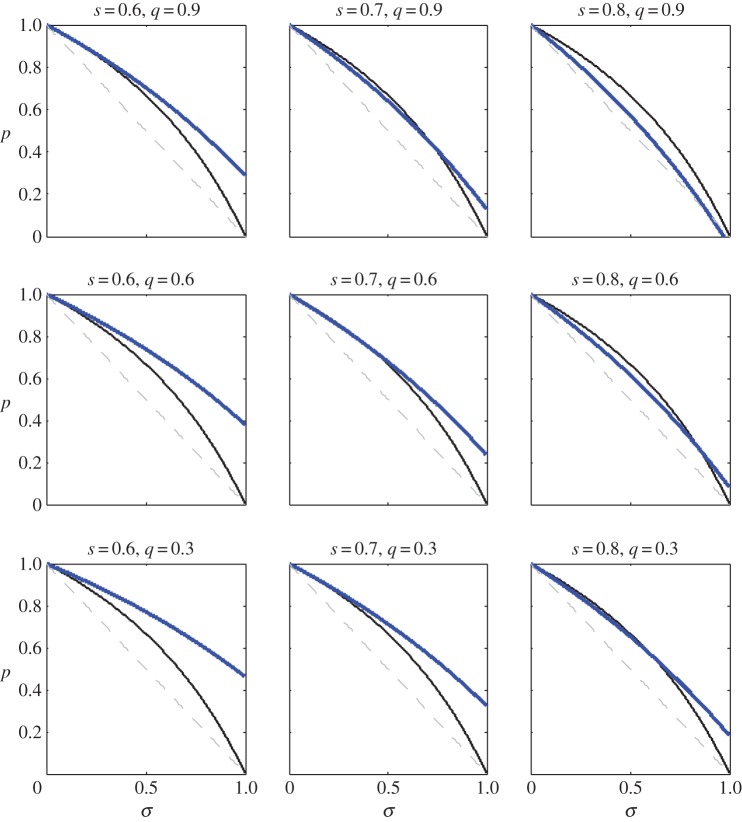
The region of ‘normal’ parameter space, *σ* and *p*, that supports cooperative breeding for various values of ‘deviant’ parameters *s* and *q*. Area above the blue curve corresponds to a selective advantage for helping; i.e. the expression in (3.2) is positive. For comparison, we have superimposed the blue curve on the relevant information from figure 1. Recall that, in order for 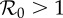 in model II, *σ* and *p* must fall above the solid black curve (*t* = 0.5 shown). In addition, *R*_0_ > 1 implies that the pair (*σ*, *p*) occurs above the dashed line. Overall, we see that increasing *s* or *q* (or both) increases the size of the region of parameter space that supports cooperative breeding. We also see that the size of the region supporting cooperative breeding in this figure is larger than that supporting cooperative breeding in model I (cf. figure 2).

When this expression is positive (respectively, negative), delayed dispersal is favoured (respectively, disfavoured) by selection. In model II, we again find that, for fixed values of *σ* and *p*, increasing ‘deviant’ survival parameters *q* and *s* increases the advantage of helping (figure 4). By comparing corresponding panels of figures 2 and 4, we also find that the set of *σ* and *p* parameter values that support the emergence of helping is larger in model II than it is in model I.

## 4. Individual-based simulation

We used individual-based simulation (*sensu* [23]) to confirm that our inclusive-fitness-based predictions were robust to changes in our assumptions about weak selection, and those about the determinism of the system as a whole. Simulations were carried out using Matlab (www.mathworks.com), and copies of the scripts used can be found in the electronic supplementary material.

In our simulations, each individual (from a finite set of individuals) has two copies of a dispersal gene. Each gene copy is simply a number between 0 and 1, and an individual's dispersal phenotype is the average of these two numbers. As one might expect, one of the genes carried by an individual is chosen uniformly at random from that individual's mother; the other gene is chosen uniformly at random from the individual's father. In order to ensure continued genetic variation in our simulations, transmission of genes from parent to offspring includes the opportunity for mutation. It is important to note that, at any time, the simulated population may consist of a variety of phenotypes—not simply wild-type individuals and a phenotypically similar mutant. It is the possibility for broad genotypic/phenotypic diversity that implies selection is no longer weak, by assumption.

Our simulations follow one or the other set of life-history events given above (model I or II), with two important changes. First, they are stochastic. Second, because the simulations allow a non-zero-level helping (i.e. incomplete dispersal) to arise, they track the number of floaters, the number of breeders without help and the number of breeders with help. Floaters still become breeders at a probabilistic rate that decreases with the total density of breeders, but in simulations the total density of breeders is the sum of the densities of breeders without help and with help.

**Figure 5.**
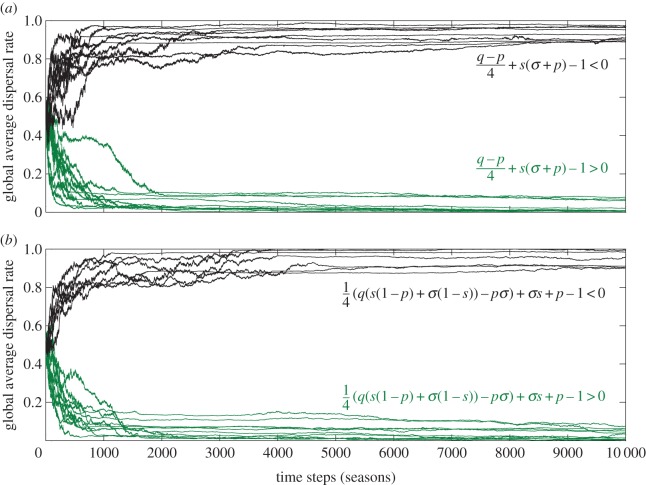
Simulation of the evolution of dispersal rate for (*a*) model I and (*b*) model II. Plots show how the mean-average dispersal rate changes over time. Recall that complete dispersal corresponds to a population in which no individual helps, and complete philopatry corresponds to a population in which every individual helps. In (*a*), black (respectively, green) trajectories show that when line (3.1) is negative (respectively, positive) the population tends towards complete dispersal (respectively, philopatry). In (*b*), black (respectively, green) trajectories show that when line (3.2) is negative (respectively, positive) the population tends towards complete dispersal (respectively, philopatry). Note that populations never exhibit complete dispersal or complete philopatry, because simulations incorporate mutation as well as a drift. Parameter values used to generate trajectories have been included with the Matlab simulation code.

Figure 5*a,b* shows simulated evolutionary trajectories for models I and II, respectively. For both models, we typically find that when the inclusive-fitness analysis predicts helping will be advantageous (i.e. when expression (3.1) is positive in model I, or when expression (3.2) is positive in model II), the average level of dispersal tends to very low levels (i.e. the population exhibits a high degree of helping). By contrast, when the inclusive-fitness analysis predicts that helpers will be at a disadvantage, the average level of dispersal becomes rather high over time. Of course, there are cases in which the action of selection is swamped by drift (data not shown), but we found these cases coincided with parameter sets that produced small simulated population sizes or parameter sets that resulted in only a slight selective advantage/disadvantage for helpers. Overall, then, we concluded that predictions from simulations matched those made by the inclusive-fitness analysis.

## 5. Discussion

### (a) Evolution of delayed dispersal and helping

We have obtained two expressions for the change in inclusive fitness that results from delayed natal dispersal and helping. We have also used individual-based simulation to show that, when the inclusive-fitness change is positive, a transition from solitary breeding to cooperative breeding occurs.

Our first expression for inclusive-fitness change was equivalent to one previously developed by Pen & Weissing [11]. It was based on a model in which normal offspring are unable to condition dispersal on parental survival. Of course, an inability to modify dispersal to respond to local breeding opportunities may simply be owing to the fact that natal dispersal occurs prior to any parental mortality event. In any event, territory inheritance in the first scenario is only possible for those offspring who also opt to help.

Our second expression for inclusive-fitness change corresponded to a model in which normal offspring disperse only when there is no opportunity to take over a territory vacated following parental death. Consequently, the second expression included a term that described the effect of local interactions among kin—a term not found in the expression developed by Pen & Weissing [11]. We found that it is possible for local interactions among kin to yield an inclusive-fitness benefit, for example when the help received by an offspring is outweighed by any risks that might come from that offspring being displaced from its birthplace. However, we also found the region of parameter space over which these net benefits might be realized to be quite small. It is more likely, in our view, that the local interaction among kin be considered an additional cost of helping.

Overall, our second result effectively extends the findings of Leggett *et al.* [13] to the case where a dominance hierarchy exists among siblings (actually, half-siblings). Our second result also supports Gaston's [12] long-standing claim that, without the possibility of territorial expansion, cooperative breeding can coincide with severe competition among relatives. Future work could certainly consider the effect of territorial expansion, and based on recent work on helping in a homogeneous population [24] we expect that such a modification would simply promote the emergence of cooperative breeding. For now, though, our second result puts Gaston's idea into a clear theoretical context.

Surprisingly, we found that, despite the occurrence of kin competition, helping in model II was predicted to occur under a broader range of ecological conditions than it was in model I (we stress that this is entirely consistent with our claim that kin competition in the second model is, most often, costly). Broader support for helping in model II occurs for two basic reasons. First, the fact that territory inheritance did not require help in model II also meant that a normal population could achieve stable numbers under a broader range of ecological conditions. In other words, there were simply more normal populations available to be invaded under model II. Second, the net benefit of helping in model II is simply larger than it was under model I. Of course, this second point is rather facile, and luckily we can point to one key fitness driver: the mortality cost of helping. As we have seen, a helper who dies in model I reduces its direct fitness. A helper who dies in model II also reduces its direct fitness, however, the reduction is countered by the fact that the sibling who benefited from the helper's actions can still inherit the territory should it become vacant. The effect of kin competition, here, is similar to that found in a theoretical study of bacterial persistence [25]. Bacterial persistence is a helpful trait, because it is associated with reduced growth rates. Importantly, modelling has revealed that kin competition can promote helpful persistence by reducing the cost of reduced growth rate (i.e. costs of entering into a quiescent state) [25].

In both model scenarios considered here, the decision to help was perfectly coupled with the decision to remain on the natal site. In other words, individuals who chose to delay dispersal also chose to help in raising half-siblings. This assumption is common to other models of cooperative breeding [11,13,14] and reflects the biology of many cooperatively breeding birds and mammals [26,27]. That said, the biology of species such as the green jay (*Cyanocorax yncas*), the Australian magpie (*Gymnorhina tibicen*) and the Siberian jay (*Perisoreus infaustus*) shows that philopatry need not imply that a non-breeder is willing to help its breeding parent [26,28]. By setting *q* = *p* in our model, one can easily turn expressions (3.1) and (3.2) into expressions for the advantage of delayed dispersal in the absence of helping. In this way, our study can also be viewed as an extension of existing work on the evolution of dispersal—work that has expressly neglected complications arising from competition among relatives [19,29]. It should be noted, however, that linking dispersal and helping, as we have done here, is known to underestimate the importance of ecological conditions (e.g. limited available habitat) in the emergence of help [30].

Our model scenarios also assume that sex allocation decisions—in other words, decisions about the effort put into reproduction through male versus female function—do not evolve. However, there is both empirical [31] and theoretical evidence [32,33] that suggests a greater fraction of a breeder's effort should be allocated to the more helpful sex. In the absence of local competition among kin, we expect cooperative breeding will emerge more readily than predicted by our inclusive-fitness analysis if breeders are able to invest more in female function. With local competition, however, predictions concerning the effect of changing sex allocation patterns become less clear. Modelling has shown that, when the more helpful sex is overproduced, local competition among kin can actually increase [34]. This may work against the emergence of cooperative breeding, but perhaps shifts towards investment in the less helpful sex could mitigate the effects of increased competition. We plan to address the topic of sex allocation and helping in the face of local competition in greater detail in the near future.

### (b) Advantage of inclusive-fitness-based theory

The results presented above can be derived using detailed mathematical models (see the electronic supplementary material), but our inclusive-fitness-based approach allowed us to develop them in a less technical way. In our view, the presentation given above highlights the importance of Hamilton's notion of inclusive fitness to sociobiology. Economic analogies are widely used to convey the adaptive significance of individual behaviour [35], and inclusive fitness provides us with the intuition we need to extend such analogies to social behaviours. As we have illustrated here, the idea that social behaviour functions to maximize an individual's inclusive fitness [36] effectively provides biologists with a ‘calculus’ of sorts that may be used to describe the action of selection in a rather straightforward manner.

Critics of inclusive fitness theory will likely have already noted that our analysis has made use of weak selection. The simulations showed that the conclusions of our analysis were robust to violations of the weak-selection assumption. More generally, weak selection is an approximation that makes inclusive-fitness-based analyses simple, just as weak selection makes population-genetic models simple (e.g. see ch. 7 of [37]). In an inclusive-fitness model, weak selection ensures that the distribution of deviant behaviour can be captured using measures of relatedness alone [38,39]. Abandoning weak selection does not mean that we are abandoning inclusive-fitness-based models; it only means that we are abandoning *simple* inclusive-fitness-based models [40]. We expect, therefore, that an inclusive-fitness-based ‘calculus’ could be used in future model extensions that included multiple helpers with synergistic interactions.
